# Perceived-stigma level of COVID-19 patients in China in the early stage of the epidemic: A cross-sectional research

**DOI:** 10.1371/journal.pone.0258042

**Published:** 2021-10-01

**Authors:** Bihua Lin, Guiqin Zhong, Zeyan Liang, Jianying Huang, Xiaofang Wang, Yanjuan Lin

**Affiliations:** 1 Department of Neurosurgery, Fujian Medical University Union Hospital, Fuzhou, China; 2 Department of Critical Medicine, Fujian Medical University Union Hospital, Fuzhou, China; 3 Department of Disinfection Supply Centre, Pingtan Branch of Fujian Medical University Union Hospital, Fuzhou, China; 4 Department of Nursing, Fujian Medical University Union Hospital, Fuzhou, China; Italian National Research Council (CNR), ITALY

## Abstract

**Objective:**

To investigate the perceived-stigma level of COVID-19 patients in the early stage of the epidemic and analysed related factors and correlations that affected the stigma levels.

**Methods:**

The COVID-19 patients were selected using the convenience sampling method. Perceived-stigma level was evaluated using the Social Impact Scale (SIS). Frequency was used to describe the general information and disease investigation status of COVID-19 patients; mean and standard deviation were used for describing stigma levels, Wilcoxon signed-ranks test (nonparametric test) was applied for pairwise comparison. Kruskal-Wallis non-parametric test for grade data, and Dwass-Steel-Critchlow-Fligner test for multiple comparative analysis. Multiple linear regression analysis was performed, and statistically significant indicators in single-factor analysis were included to investigate the independent factors of stigma. The *p*<0.05 was considered statistically significant.

**Results:**

SIS score of the 122 COVID-19 patients averaged 57.37±9.99 points. There were statistically significant differences in perceived-stigma levels among patients of different ages (*p* = 0.008), occupation (*p* <0.001), marital status (*p* = 0.009), and disease severity (*p* = 0.020). Multivariate logistic regression analysis revealed that age was the main influencing factor of stigma (*p*<0.05).

**Conclusions:**

The overall perceived-stigma level of COVID-19 patients in the early stage of the epidemic was moderate. Younger, unmarried, and severely ill patients had a higher level of perceived-stigma, with age being the main factor. More attention should be given to the young COVID-19 patients.

## Introduction

On January 7, 2020, the Chinese Center for Disease Control and Prevention identified and isolated a new type of coronavirus and Coronaviridae Study Group of the International Committee on Taxonomy of Viruses named it severe acute respiratory syndrome coronavirus 2 (SARS-CoV-2) [1–3]. By the end of January 2020, the new coronavirus epidemic had spread rapidly, causing widespread global concern [4]. The main manifestations of coronavirus disease 2019 (COVID-19) are fever, dry cough, and fatigue, and respiratory droplets and close contact are the main routes of transmission [5]. On March 11, 2020, the World Health Organization announced that COVID-19 had become a pandemic, and 114 countries reported more than 118,000 cases [6]. The pandemic has not just been a medical phenomenon, it has also affected individuals and societies and caused division, anxiety, stress, stigma, and xenophobia [7].

Around the world, the public was informed about the physical effects of COVID-19 infection and the steps to be taken to prevent exposure to the coronavirus and manage COVID-19 [8]. Most of the efforts have been focussed on understanding the epidemiology, clinical characteristics, transmission patterns, and management of COVID-19 [9–11]. At the same time, many scholars have called for learning from past pandemics and paying attention to the risk of stigmatization of COVID-19 patients [12–15]. Previous studies have found that patients suffering from serious infectious diseases, such as severe acute respiratory syndrome (SARS) and acquired immunodeficiency syndrome (AIDS) are common targets of discrimination [16, 17]. During the COVID-19 pandemic, infected patients were arguably the group most vulnerable to stigmatisation and mental health issues [18, 19]. In HIV/AIDS and tuberculosis patients, stigma has been shown to be positively correlated with depression [17, 20]. Furthermore, the fear of being stigmatised can delay the diagnosis of infected persons and/or lead to them not being identified/diagnosed [21]. The psychological problems in turn may alter their attention and decisioning capability which is not only limited to affect their mental wellbeing but can also affect in managing the ongoing crisis [22]. Therefore, as healthcare providers, we must understand the possibility and severity of stigmatisation of people infected with SARS-CoV-2.

In addition, stigma must be evaluated from multiple perspectives (perceived-stigma, affiliate stigma, public stigma) so that interventions aiming to reduce stigma can target each condition from multiple levels [23]. Public stigma represents the prejudice and discrimination directed at a group by the population [24]. Perceived-stigma comes into being when people internalize these public negative attitudes and suffer numerous negative consequences as a result [25]. Perceived-stigmatization diminishes feelings of self-worth, such that the hope in achieving goals is undermined [26]. This study aimed to investigate the level of perceived-stigma towards COVID-19 patients in the early stage of the epidemic and analysed related factors and correlations that affected the stigma levels.

## Materials and methods

### Patients

Convenience sampling was used in this study. From February 27th to March 12th, 2020, two hospitals in Wuhan, China, that admitted patients with COVID-19 were selected as research sites. The inclusion criteria were as follows: (1) meeting the criteria of the diagnosis and treatment plan (the seventh edition of the trial [27]) for confirmed COVID-19 cases; (2) being able to independently cooperate to complete the investigation; and (3) providing verbal informed consent to participate in this study (for minors, obtaining consent from both them and parents at the same time); Exclusion criteria were: (1) concomitant, serious organic or physical diseases (eg. epilepsy, hypertension, diabetes etc.); (2) past history of mental illness (eg. schizophrenia, depression etc.); and (3) inability to speak Chinese. This study adopted the online questionnaire survey method. After providing verbal informed consent, the patient was sent the questionnaire via WeChat Official Account, and they filled it out anonymously on their cell phone. This study has been granted by the Ethics Committee of Fujian Medical University Union Hospital, Fuzhou, China (2020 KY0154).

### Data collection

The baseline information of the patients was collected through a self-designed general information questionnaire and included data on gender, age, education level, economic status, marital status, occupation, residence, critical illness, etc. Participants’ perceived-stigma levels were assessed using the Social Impact Scale (SIS; Cronbach’s α 0.85–0.90) [28, 29], which is a self-report questionnaire that measures the effect on patients and their families of negative social attitudes toward the patients’ health or mental health condition, including social isolation, internal shame, social exclusion, and economic insecurity. There were 24 items in 4 dimensions, and each item was scored using a Likert 4-level method. There were 4 options: strongly agree, agree, disagree, and strongly disagree. The total score was 96 points.

### Statistical analysis

This study used SPSS23.0 (IBM, Corp., Armonk, NY, USA) software for data analysis. Frequency was used to describe the general information and disease investigation status of COVID-19 patients; median mean and standard deviation were used to describe the perceived-stigma level of COVID-19 patients. Wilcoxon signed-ranks test (nonparametric test) was applied for pairwise comparison. Kruskal-Wallis non-parametric test was applied for grade data, and Dwass-Steel-Critchlow-Fligner (DSCF) test for multiple comparative analysis; multiple linear regression analysis was used, and statistically significant indicators in single-factor analysis were included to further examine the independent influencing factors of stigma in COVID-19 patients. All statistics were tested using a two-sided test, and *p*<0.05 was considered statistically significant.

## Results

### General characteristics

A total of 125 questionnaires were distributed in this study; 122 valid questionnaires were returned, and the effective response rate was 97.6%. Among the 122 patients, 59.8% were male and 40.2%, female; 52.5% were 50 years and younger, and 47.5% were 50 years and older. Among the 122 patients, 90% were within 1 week from onset to hospitalisation, 47.5% were severe COVID-19 patients, and 52.5% were mild COVID-19 patients (Table 1).

**Table 1:** Survey results of stigma among COVID-19 patients with different characteristics.

Items	Numbers	Ratio	SIS* for Stigma Median (Interquartile, Range)	*χ* ^*2*^ */Z*	*p* values
Gender					
Male	73	59.8%	58(52,64)	-1.362^#^	0.173
Female	49	40.2%	55(51.5,59)		
Age					
≤50 years	64	52.5%	58(53,64)	-2.638^#^	**0.008**
>50 years	58	47.5%	55(49.75,58.25)		
Education					
Primary	21	17.2%	54(48.5,57.5)	6.038	0.110
Junior	24	19.7%	56(50.25,60)		
Senior	26	21.3%	56.5(53,65.25)		
Collegial	51	41.8%	58(53,64)		
Occupation					
Migrant workers/farmers	32	26.2%	52(47.25,56)	18.530	**<0.001**
Civil servants	27	22.1%	58(54,62)		
Business/freelancing	49	40.2%	58(53,63)		
Students	14	11.5%	62(52,69)		
Marital status					
Married	98	80.3%	56(52,61.25)	9.435	**0.009**
Unmarried	11	9.0%	59(53,84)		
Divorced/widowed	13	10.7%	52(43,57)		
Financial Situation					
No difficulty at all	24	19.7%	56.5(52,60)	0.089	0.993
Well enough	67	54.9%	56(52,61)		
A little difficult	26	21.3%	57(49.75,64)		
Very difficult	5	4.1%	56(53,59.5)		
Wuhan residents					
Yes	116	95.1%	56(52,60)	0.302^#^	0.762
No	6	4.9%	56.5(51.75,65.25)		
Registered residence					
Village	2	1.6%	61(51,-)	0.150	0.928
Town	12	9.9%	55(52.25,58.75)		
City	108	88.5%	56(52,60.75)		
Severity of disease					
Severe case	58	47.5%	56(53,67)	2.333^#^	**0.020**
Mild case	64	52.5%	56(49,59)		
Family infection status					
Yes	59	48.4%	55(50,62)	-1.182^#^	0.237
No	63	51.6%	57(53,60)		
Time between diagnosis and hospitalization					
≤1 week	100	90.0%	55.5(52,60)	1.690^#^	0.091
>1 week	22	10%	58.5(54,65)		

*SIS, Social Impact Scale;

^#^ Z value.

### SIS scores for perceived-stigma

The SIS total score of COVID-19 patients averaged 57.37±9.99 points, which was generally at a moderate level.

### Single-factor analysis results

The results are as follows: gender (*p* = 0.173), education level (*p* = 0.110), economic situation (*p* = 0.993), whether the patient is a resident of Wuhan (*P* = 0.762), family residence (*p* = 0.928), and whether the patient’s family is infected (*p* = 0.237). The time between diagnosis and hospitalization (*p* = 0.091) had no significant association with the level of perceived-stigma (*p*>0.05) (see Table 1 for details). Age (*p* = 0.008), occupation (*p*<0.001), marital status (*p* = 0.009), and disease severity (*p* = 0.020) had statistically significant differences in perceived-stigma (Table 1). Further, the multiple comparison analysis of DSCF for different occupations and marital status was used to conduct a two-sided test, and pairwise comparisons were performed. The level of perceived-stigma of COVID-19 patients was significantly lower among migrant workers and farmers compared with that among other occupations (*p*<0.05) (Table 2). Divorced or widowed patients with COVID-19 had more severe perceived-stigma (*p*<0.05) (Table 3) than married patients.

**Table 2:** Influence of different occupations on stigma of COVID-19 patients.

Sample 1—sample 2	*Z* values	DSCF values	*p* values
Business/freelancing- Students	-1.374	1.943	0.516
Business/freelancing- Civil servants	-0.332	0.469	0.987
Business/freelancing- Migrant workers/farmers	3.110	4.398	**0.010**
Students- Civil servants	1.226	1.734	0.610
Students- Migrant workers/farmers	3.480	4.921	**0.003**
Civil servants- Migrant workers/farmers	3.508	4.961	**0.003**

**Table 3:** Influence of different marriages on stigma of COVID-19 patients.

Sample 1—sample 2	*Z* values	DSCF values	*p* values
Married- Unmarried	-1.848	2.614	0.154
Married- Divorced/widowed	2.351	3.325	**0.049**
Unmarried- Divorced/widowed	2.609	3.690	**0.025**

### Multivariate analysis results

The total perceived-stigma was divided into dependent variables, and the age, marital status, severity of illness, and occupation had a significant impact on the stigma level of COVID-19 patients. Four factors were determined as independent variables, and a multiple linear regression analysis was performed. The results showed that age was the main influencing factor of the perceived-stigma level of COVID-19 patients (*p*<0.05), explaining 15.7% of the total variation (Table 4).

**Table 4:** Multiple linear regression analysis of stigma in COVID-19 patients.

Independent variables	Non standardized coefficient	Standardized coefficient Beta	*t*	*P*	Adjusted R^2^	F	*p*
(Constant)	64.718		12.247	**<0.001**	0.157	6.645	**<0.001**
Severity of disease	-2.869	-0.144	-1.592	0.114			
Age	-0.144	-0.220	-2.149	0.034*			
Occupation	1.916	0.191	1.984	0.050			
Marriage	-0.269	-0.018	-0.208	0.836			

## Discussion

The tension caused by the COVID-19 pandemic has turned into a crisis with unprecedented consequences globally. The impact of the pandemic and the adopted quarantine measures on our mental health is obvious. However, few studies have provided significant evidence to explain its impact on the mental health of COVID-19 patients. This study investigates the perceived-stigma status of SARS-CoV-2 infected persons in epidemic areas, analyses the relevant factors and correlations that affect the stigma levels, and provides a basis for clinical decision-making.

The results of this study show that the average stigma score of COVID-19 patients participating in the survey was in the moderate level. This is similar to the results of research by Lee et al. [30] showing that 87.4% of respondents reported both psychological and somatic distress following the outbreak of SARS due to stigma. The transmission index of influenza is close to 1.5, that of SARS is 2–2.5, and COVID-19 is close to 3. The rapid transmission and overwhelmed hospitals/ICUs of COVID-19 has aggravated public fear [31]. With the increase in the number of patients, the public’s feelings of fear, rejection, and isolation of COVID-19 patients has gradually become stronger and more widespread [32]. In addition, the uncertainty in the early treatment of the disease has led to impaired patient confidence in recovery and decreased compliance with treatment. Centralised isolation treatment indirectly defines COVID-19 patients as a distinctive risk group. The interaction of multiple factors has led to a high stigma in patients with early COVID-19. Many studies have shown that older people have a more positive attitude than younger people [33, 34]. The results of these studies suggest that the level of stigma of COVID-19 patients under 50 years of age is significantly higher than that of patients over 50 years of age (*p*<0.05). This may be because younger patients have a number of social functions and need to shoulder important responsibilities for supporting family members; they worry that social barriers caused by the illness will affect the overall state of the family. On the other hand, older patients have relatively fewer responsibilities towards the family. In addition, their rich life experience helps them maintain a more stable mentality while facing the disease. In addition, the results of the multiple linear regression analysis also indicate that age is an independent influencing factor of stigma in COVID-19 patients. Therefore, we should pay more attention to the stigma status of young and middle-aged COVID-19 patients and provide targeted anti-stigma interventions [35, 36]. For instance, government and hospital authorities should make operational strategies to provide mental healthcare for the quarantined young individuals [33]. However, Yuan et al. conducted a study to compare differences in perceived stigma between COVID-19 survivors and healthy controls, and the results didn’t suggest the effect of age on the level of stigma [37].

COVID-19 patients with severe disease may have a higher SARS-CoV-2 load; more severe cough, sputum, and other symptoms; and higher infectivity. Further, patients with severe COVID-19 symptoms may receive more information from friends, family members, news, and electronics regarding the disease. Social isolation and exclusion reported by the media may, thus, have stronger effects on these patients. Therefore, compared to patients with mild COVID-19, patients with severe COVID-19 may have a higher perceived-stigma.

The type of occupation also affects the level of stigma. The perceived-stigma of COVID-19 patients was the lowest among migrant workers and farmers, followed by that among company employees or freelancers, and was highest among students. The student group is at a sensitive phase of individual physical and mental development. Once the illness spreads among classmates, the other classmates and teachers are readily isolated; this instils the fear that the illness will affect employment prospects and social communication. Further, the state of psychological and emotional instability among students and weakens their ability to bear the mental trauma. Once they are excluded or isolated, they are more likely to experience negative emotions [38]. Therefore, it is necessary to strengthen the regular mental health assessment of the student group among COVID-19 patients, with a focus on early psychological intervention.

The results also show that divorced, widowed, or unmarried COVID-19 patients experience more perceived-stigma. This is related to the lack of spousal care, loss of emotional support, lack of people to talk to, insufficient family support, and stronger stigma experience. Married patients, on the other hand, have a complete family structure providing more care, which results in the patient being happier, actively facing the disease, and experiencing reduced degree of stigma. Moreover, Mukerji et al. studied the stigma characteristics of patients with tuberculosis and found that the loss of marital prospects was the commonly reported concern among younger, unmarried participants. And their marriage had been called off once their family found out they were sick [39].

Person et al. [40] investigated the stigma of patients during SARS in China in 2003 and believed that stigma appeared during the outbreak. The COVID-19 outbreak is similar to that of SARS. At the first signs of an outbreak, strategies to eliminate or reduce stigma should begin simultaneously [41, 42]. Choosing to conduct evaluation and intervention at the first contact with the patients can reduce adverse effects caused by stigma. At the national and government levels, having better social support can reduce the stigma level of patients [43, 44]. During the outbreak of the COVID-19 epidemic, China introduced a policy of free screening and free treatment of new coronavirus-related pneumonia for its citizens, which reduced the financial burden for patients. In addition to increasing the rate of consultation for patients, it also reduced the financial burdens of COVID-19 patients with pneumonia. At the social level, in addition to the scientific popularisation of the spread of COVID-19 among the public and implementation of personal protective measures during the epidemic, government personnel and news media should also popularise the curability of COVID-19 and reduce the risk of unnecessary isolation, disgust, and stigma for COVID-19 patients. Medical and health institutions should perform regular mental health follow-ups (including assessment of stigma level) for recovered COVID-19 patients after discharge [45].

This study is the first to quantitatively describe the perceived-stigma level of COVID-19 patients in the early stage. The population investigated was from the centre of the epidemic in China, which could reflect the perceived-stigma towards COVID-19 patients to a certain extent. However, there are several limitations in our study: (1) The sample size was not large and the scope of the investigation was limited. (2) The study investigated patients’ perceived-stigma levels during the hospitalisation period only. (3) The SIS was only used to assess stigma among HIV/AIDS and cancer patients. (4) The research was a cross-sectional study, lack of control group and follow-up results. (5) The study used convenience sampling. Hence, there may be potential bias for the accuracy of the results. Nonetheless, historically, infectious diseases have faced the most powerful stigma among public health concerns, and the study still sought to expose the degree of discrimination and prejudice suffered by those infected in the early stages of the epidemic. Future research with larger sample sizes in design is necessary to understand the underlying adaptations and to better evaluate the individual variability.

## Conclusions

The overall perceived-stigma level of COVID-19 patients in the early stage of the epidemic is at a moderate to severe level. Age, marital status, occupation, and disease severity may affect the perceived-stigma level of patients. Patients who are young, unmarried, and severely ill have a higher level of perceived-stigma. Age is an independent factor that affects the perceived-stigma level of COVID-19 patients. Further, it is necessary for psychiatric organizations and other medical organizations collaborate with each other and develop clinical consensus guidelines to reduce the perceived-stigma level of COVID-19 patients.

## Supporting information

S1 File The file contains the Social Impact Scale (English version). (DOCX)

S2 File The file contains the Social Impact Scale (Chinese version). (DOCX)

S3 File The file contains the raw data used for Tables 1–4. (XLSX)
