# Supplementary material for: Perceived-stigma level of COVID-19 patients in China in the early stage of the epidemic: A cross-sectional research
Source: PLoS One. 2021 Oct 1;16(10):e0258042. doi: 10.1371/journal.pone.0258042 (PMC8486130; doi:10.1371/journal.pone.0258042)
Supplement: S1 File — (DOCX) [file pone.0258042.s001.docx]

**COVID-19 Patient Stigma Questionnaire**Dear patients:

We sincerely invite you to participate in this survey. The purpose of this survey is to understand the effects of COVID-19 patients on social exclusion, economic discrimination, intrinsic shame and social isolation, so that we can better carry out comprehensive care for COVID-19 patients and promote physical and mental health.The survey is conducted on a voluntary basis. Your personal information will be kept strictly confidential and used for research and scientific analysis only. Except for members of the research team, others have no right to read it.Thank you for your cooperation and wish you a speedy recovery!

**General situation survey**
Name: Hospital: Medical Record No.:

Date of filling:
1. Gender: ① Male ② Female
2. Age:
3. Mobile phone number:

4. Education: ① primary school or below ② junior high school ③ high school or technical secondary school ④ junior college or above
5. Occupation: ① migrant worker or farmer ② civil servant or public institution ③ enterprise or freelancer ④ student
6. Are you a resident of Wuhan? ① Yes ② No
If you are not a resident of Wuhan, you will come to Wuhan for the following purposes: ① travel ② visit relatives ③ work ④ other purposes
7. Family residence: ① rural area ② town ③ city
8. Economic situation: ① no difficulty at all ② OK ③ a little difficult ④ very difficult.
9. Marital status: ① Married ② unmarried ③ divorced or widowed

**Disease investigation**10. Date of diagnosis:

11. Duration of first hospitalization:

12. Do you have a family member infected with COVID-19? ① Yes(How many family members are infected : ) ② No
13. If you have a family member who is also infected with COVID-19, are you now cured and discharged from hospital? ① Yes ② No

**Social Impact Scale**Please tick "√" according to your true feelings in the last two weeks.

1. I had financial difficulties that affected the way I felt about myself.
① Strongly agree ② Agree ③ Disagree ④ Strongly disagree

2. My illness affects my job stability.
① Strongly agree ② Agree ③ Disagree ④ Strongly disagree

3. My boss or colleagues discriminate against me.
① Strongly agree ② Agree ③ Disagree ④ Strongly disagree

4. My personal relationships have suffered because of financial difficulties.
① Strongly agree ② Agree ③ Disagree ④ Strongly disagree

5. Some people act as if I'm less competent than usual.
① Strongly agree ② Agree ③ Disagree ④ Strongly disagree

6. My illness makes me feel more disrespected than usual.
① Strongly agree ② Agree ③ Disagree ④ Strongly disagree

7. I feel isolated from healthy people.
① Strongly agree ② Agree ③ Disagree ④ Strongly disagree

8. I feel that other people are worried that they will catch my illness from touching me, such as shaking my hand or eating the food I prepare.① Strongly agree ② Agree ③ Disagree ④ Strongly disagree

9. I felt that people shunned me because of my illness.
① Strongly agree ② Agree ③ Disagree ④ Strongly disagree

10. Some family members rejected me because of my illness.
① Strongly agree ② Agree ③ Disagree ④ Strongly disagree

11. I feel that others think I am to blame for my illness.
① Strongly agree ② Agree ③ Disagree ④ Strongly disagree

12. I don't feel I can admit my illness to others.
① Strongly agree ② Agree ③ Disagree ④ Strongly disagree

13. I worry about people telling people about my illness without my permission.
① Strongly agree ② Agree ③ Disagree ④ Strongly disagree

14. I feel I need to keep my illness a secret.
① Strongly agree ② Agree ③ Disagree ④ Strongly disagree

15. I feel that some friends reject me because of my illness.
① Strongly agree ② Agree ③ Disagree ④ Strongly disagree

16. More than usual, I need to make sure that people care.
① Strongly agree ② Agree ③ Disagree ④ Strongly disagree

17. I feel lonely more often than usual.
① Strongly agree ② Agree ③ Disagree ④ Strongly disagree

18. Because of my illness, I feel like I'm in an unequal position in my relationships.
① Strongly agree ② Agree ③ Disagree ④ Strongly disagree

19. I feel that I am at least partly to blame for my illness.
① Strongly agree ② Agree ③ Disagree ④ Strongly disagree

20. I feel my abilities are worse than they were before I got sick.
① Strongly agree ② Agree ③ Disagree ④ Strongly disagree

21. I've had some embarrassing situations because of my illness.
① Strongly agree ② Agree ③ Disagree ④ Strongly disagree

22. Because of my illness, I seem to feel embarrassed and nervous when people are around me.
① Strongly agree ② Agree ③ Disagree ④ Strongly disagree

23. Sometimes I feel useless because of my illness.
① Strongly agree ② Agree ③ Disagree ④ Strongly disagree

24. The change in my appearance has affected my social relationships.
① Strongly agree ② Agree ③ Disagree ④ Strongly disagree
