# Supplementary material for: Perceived-stigma level of COVID-19 patients in China in the early stage of the epidemic: A cross-sectional research
Source: PLoS One. 2021 Oct 1;16(10):e0258042. doi: 10.1371/journal.pone.0258042 (PMC8486130; doi:10.1371/journal.pone.0258042)
Supplement: S2 File — (DOCX) [file pone.0258042.s002.docx]

**COVID-19患者病耻感调查表**

尊敬的病友，您好。我们诚挚邀请您参加本次的调查，本次调查的目的是为了解COVID-19患者在社会排斥、经济歧视、内在羞耻感和社会隔离中的影响效果，以便我们更好地开展对COVID-19患者的全方面的护理，促进身心健康。该调查是建立在自愿原则上，您的个人信息将会绝对保密，仅用于研究和科学分析，除课题组成员可以阅读外，其余人员无权阅读。谢谢您的配合，祝您早日康复！

**一般情况调查**

**姓名： 所在医院： 病案号： 填表日期：**

1.性别：①男 ②女

2.年龄： 岁

3.手机号码：

4.受教育程度：①小学及以下 ②初中 ③高中或中专 ④大专及以上

5.职业： ①民工或务农 ②公务员或事业单位 ③企业或自由职业者 ④学生

6.是否为武汉居民：①是 ②否

如果非武汉居民，此次来武汉目的是：①旅游 ②探亲 ③工作 ④其他

7.家庭居住地：①农村 ②乡镇 ③城市

8.经济状况：①完全无困难 ②还可以 ③有点困难 ④非常困难。

9.婚姻情况：①已婚 ②未婚 ③离异或丧偶

**疾病情况调查**

10.确诊时间： 月 日

11.第一次住院时间： 月 日

12.您是否有家人感染了新冠肺炎：①是（ 个） ②否

13.如果您有家人也感染了新冠肺炎，目前是否痊愈出院：①是 ②否

**社会影响量表**

请根据您最近二周的真实感受，选择最符合您的答案打“√”。

1.我曾因财务困难，而影响到我对自己的感受。

①极为同意②同意③不同意④极为不同意

2.我的疾病影响我工作的稳定性。

①极为同意②同意③不同意④极为不同意

3.我的老板或同事歧视我。

①极为同意②同意③不同意④极为不同意

4.我曾因财务困难，人际关系受到影响。

①极为同意②同意③不同意④极为不同意

5.有些人表现出好像我的能力比平时差。

①极为同意②同意③不同意④极为不同意

6.我的疾病让我觉得我比平常更不受尊重。

①极为同意②同意③不同意④极为不同意

7.我感觉自己被隔绝于健康人之外。

①极为同意②同意③不同意④极为不同意

8.我感觉他人会担心因为和我的接触，而感染了我的病，如与我握手或吃我准备的食物。①极为同意②同意③不同意④极为不同意

9.我感觉别人因为我的病而回避我。

①极为同意②同意③不同意④极为不同意

10.一些家人因为我的病而排拒我。

①极为同意②同意③不同意④极为不同意

11.我觉得别人认为我的病应该怪我自己。

①极为同意②同意③不同意④极为不同意

12.我觉得我无法对别人坦承我的疾病。

①极为同意②同意③不同意④极为不同意

13.我担心有人在没有我的允许下告知他人我的疾病。

①极为同意②同意③不同意④极为不同意

14.我觉得我需要为我的疾病保密。

①极为同意②同意③不同意④极为不同意

15.我感觉有些朋友因为我的病而排拒我。

①极为同意②同意③不同意④极为不同意

16.我比平常更需要确定别人对我的关心。

①极为同意②同意③不同意④极为不同意

17.我比平常更常感到孤单。

①极为同意②同意③不同意④极为不同意

18.因为我的疾病，我觉得我在人际关系中处于不平等的状态。

①极为同意②同意③不同意④极为不同意

19.我觉得我会得病至少有部分该怪我自己。

①极为同意②同意③不同意④极为不同意

20.我觉得我的能力比生病前还差。

①极为同意②同意③不同意④极为不同意

21.因为我的病我遇到一些令我难堪的状况。

①极为同意②同意③不同意④极为不同意

22.因为我的疾病，别人在我周遭时我似乎感到尴尬和紧张。

①极为同意②同意③不同意④极为不同意

23.为我的疾病，有时候我觉得自己没有用。

①极为同意②同意③不同意④极为不同意

24.我外观的改变已经影响我的社交关系。

①极为同意②同意③不同意④极为不同意
